# Autoimmune Myositis Presenting With Fulminant Rhabdomyolysis and Dialysis-Dependent Acute Kidney Injury in the Context of Nitrofurantoin Exposure and Chronic Statin Use: A Case Report

**DOI:** 10.7759/cureus.105252

**Published:** 2026-03-15

**Authors:** Saw Danny Lynn, Kaung Pyae Sone, Kyan Thein, Zin Zin Htike

**Affiliations:** 1 Acute Medicine, Nottingham University Hospitals NHS Trust, Nottingham, GBR; 2 Internal Medicine, Nottingham University Hospitals NHS Trust, Nottingham, GBR; 3 Diabetes and Endocrinology, Nottingham University Hospitals NHS Trust, Nottingham, GBR

**Keywords:** acute renal injury, autoimmune necrotizing myositis, immune-mediated necrotizing myopathy, inflammatory myositis, non-traumatic rhabdomyolysis, rhabdomyolysis with acute renal failure

## Abstract

Autoimmune inflammatory myopathies (AIM) can present insidiously or abruptly and are often indistinguishable from drug-induced myopathy. Severe rhabdomyolysis with acute kidney injury (AKI) as a complication in an elderly patient is a high-stakes presentation. We present a case of a 77-year-old woman on long-standing atorvastatin therapy who presented with an acute onset of proximal lower-limb weakness, CK > 42,000 U/L, stage 3 AKI, and transaminitis a week after a short course of nitrofurantoin. Magnetic resonance imaging (MRI) and electromyography (EMG) confirmed inflammatory myopathy; serology was positive for Mi-2α and PL-7 antibodies but negative for 3-hydroxy-3-methylglutaryl-coenzyme A (HMG-CoA) reductase antibodies. Despite statin withdrawal and cessation of nitrofurantoin and vigorous fluid resuscitation, she needed hemodialysis. High-dose intravenous methylprednisolone therapy followed by oral prednisolone resulted in clinical recovery as well as resolution of biochemical abnormalities, with CK returning to normal at discharge (189 U/L). This case demonstrates the coexistence of drug-induced myopathy and double myositis antibody positivity (anti-Mi-2α and anti-PL-7) resulting in AKI requiring dialysis, and a favorable response to corticosteroid therapy without the need for IVIG. In older adults presenting with marked CK elevation and renal failure, AIM should be considered as one of the differential diagnoses, even in the context of concurrent statin exposure. Myositis-specific antibody pattern, including the uncommon double-positive combination, serves as an important guide for diagnosis and initial immunosuppression, while drug cessation alone may not achieve adequate disease control.

## Introduction

Double seropositivity (e.g., anti-Mi-2 with anti-PL-7) is rare but recognized and may correlate with more complex phenotypes [1-4]. Autoimmune inflammatory myopathies (AIMs) are heterogeneous disorders characterized by proximal muscle weakness, myofiber inflammation/necrosis, and elevated creatine kinase (CK) levels [3,4]. The clinical spectrum overlaps with toxic myopathies and with rhabdomyolysis due to various triggers (trauma, infection, drugs). Appropriate classification integrates clinical features, magnetic resonance imaging (MRI)/electromyography (EMG), histopathology, and critically, myositis-specific/associated autoantibodies. Statins are a common confounder: they can induce self-limited myalgias, precipitate rhabdomyolysis, or trigger immune-mediated entities, including dermatomyositis (DM) and immune-mediated necrotizing myopathy (IMNM). Distinguishing these requires clinicopathologic correlation and targeted serology (e.g., anti-3-hydroxy-3-methylglutaryl-coenzyme A (HMG-CoA) reductase (anti-HMGCR) for IMNM) [3-6]. In addition, concomitant administration of certain medications can modulate myotoxic risk. Recent case reports published DM with rhabdomyolysis on background statin therapy compounded by proton-pump inhibitor and loop diuretic exposure and incomplete response to steroids/rituximab [7,8].

## Case presentation

A 77-year-old woman with a background history of hypertension, ischemic heart disease, and stable angina presented for medical admission with a one-week history of progressive bilateral proximal lower-limb weakness without associated sensory symptoms, fever, or rash. The patient denied any recent or intense physical exertion. Regular medications included low-dose aspirin, amlodipine, ramipril, bisoprolol, isosorbide mononitrate, and long-term atorvastatin 40 mg daily. She also had a five-day course of nitrofurantoin for a urinary tract infection one week before presentation.

On examination, she was afebrile and hemodynamically stable. Neurological examination revealed marked proximal lower-limb weakness with preserved distal power below the knee joints; upper limbs were spared. There were no clinical signs of systemic infection. She had no rash or other cutaneous features of DM.

**Figure 1:**
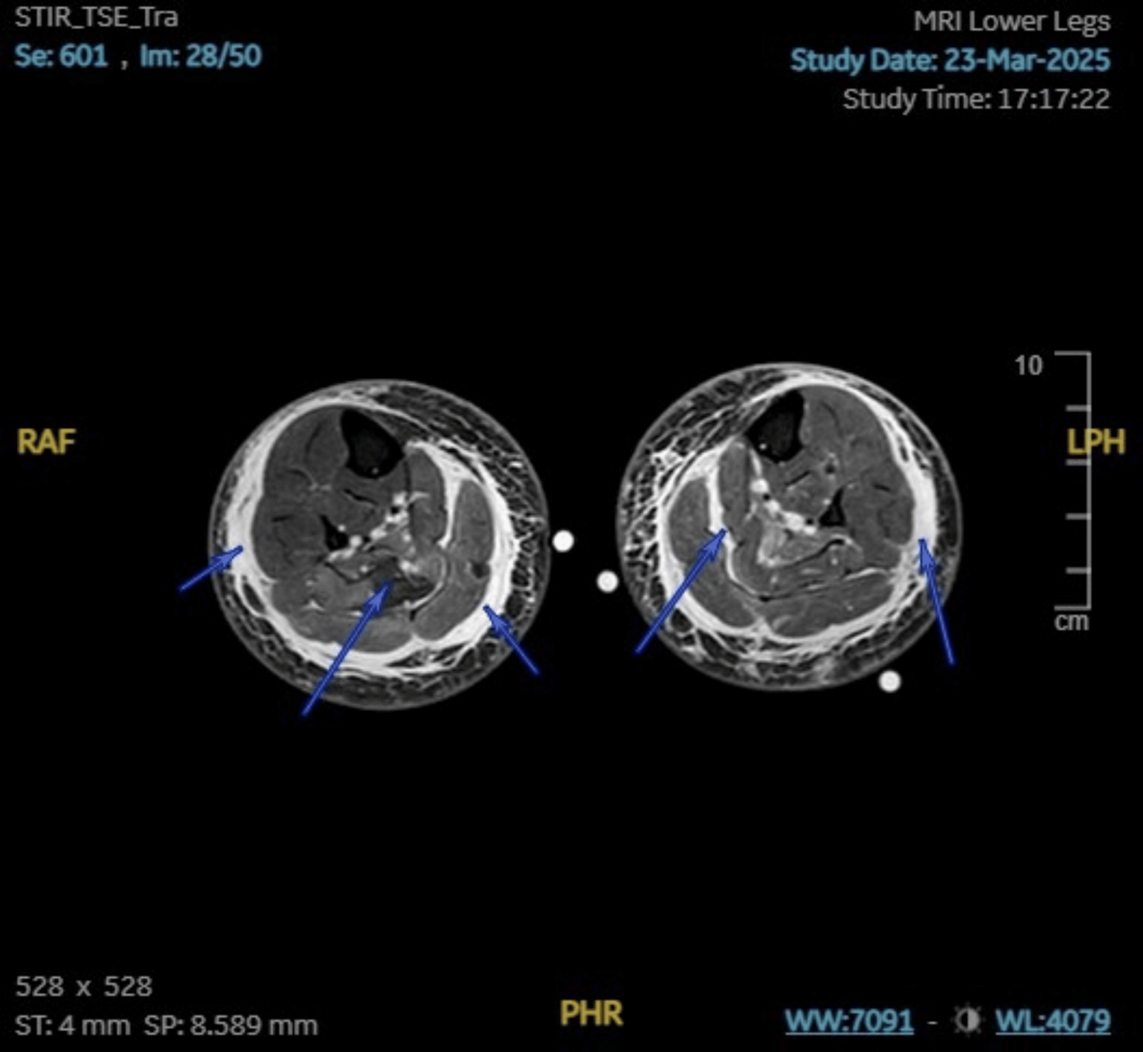
Axial short tau inversion recovery (STIR) view of the MRI of the lower limbs. Arrows indicate bilateral perimuscular and fascial soft tissue swelling of the lower limbs.

**Figure 2:**
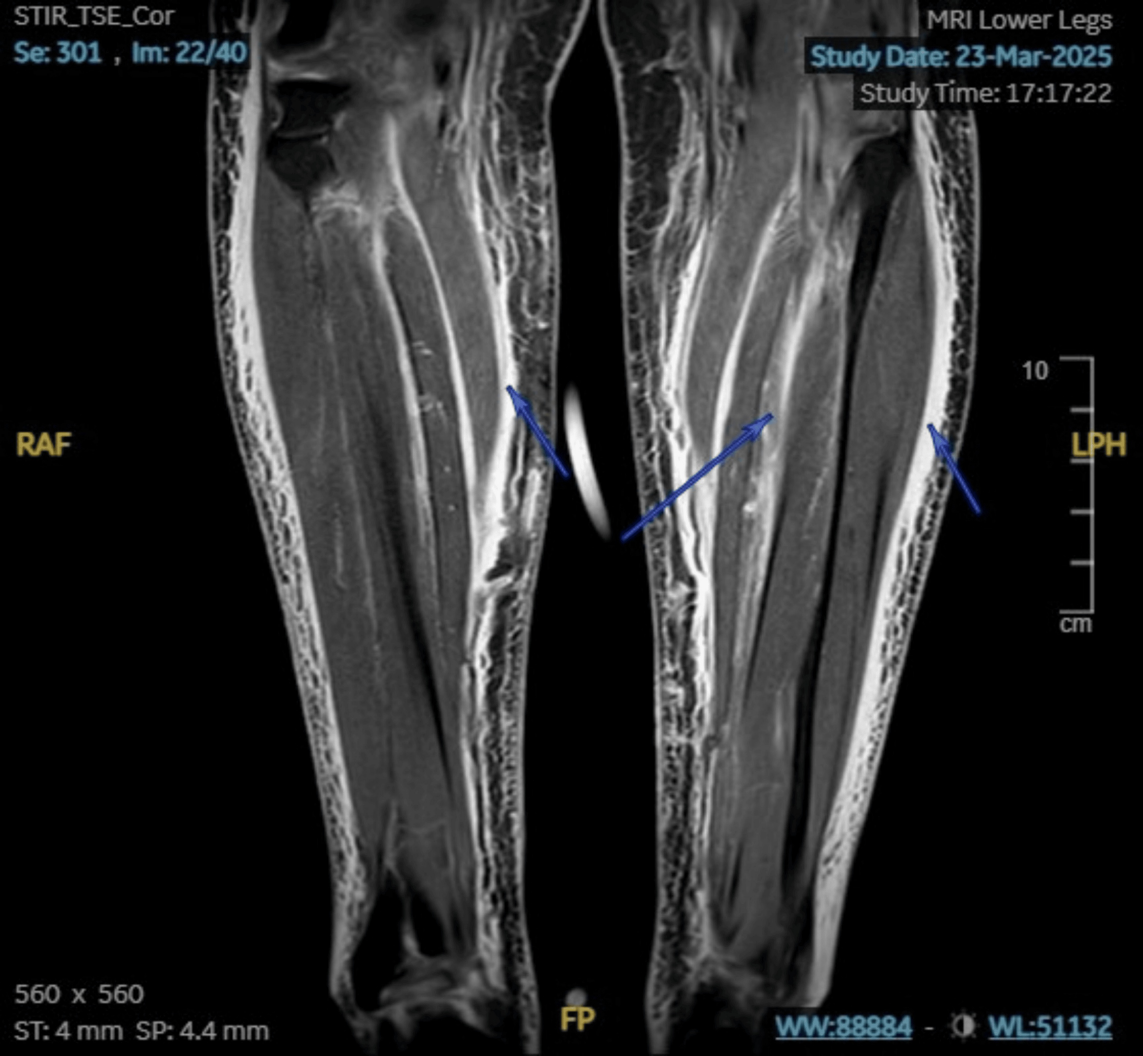
Coronal short tau inversion recovery (STIR) view of the MRI of the lower limbs.

**Figure 3:**
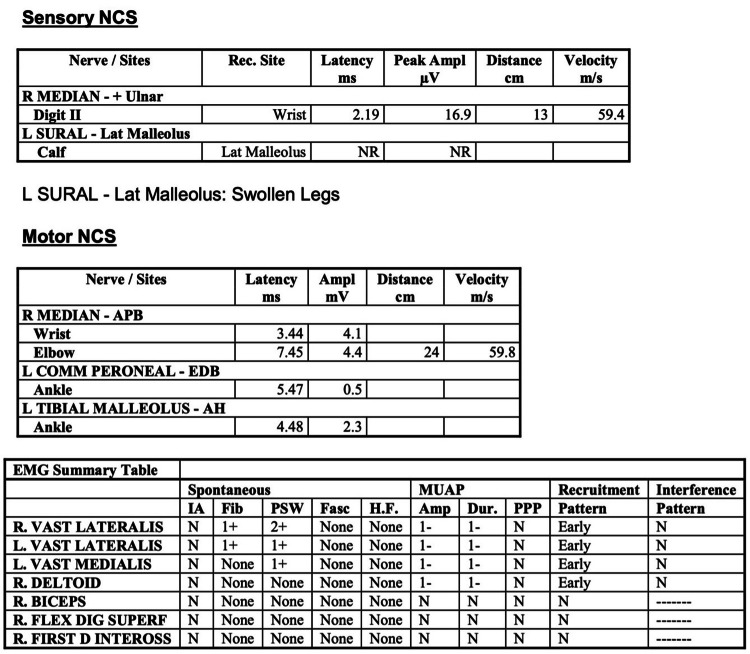
Electromyography (EMG) interpretations. The report suggests a diagnosis of myositis without features of demyelinating polyneuropathy. Image credit: Nottingham University Hospitals NHS Trust. The image is reproduced with permission and is licensed for publication under the Creative Commons Attribution (CC-BY 4.0) license.

Initial biochemical results showed CK > 42,000 U/L; creatinine 400 µmol/L (estimated glomerular filtration rate (eGFR) 9 mL/min/1.73 m²); alanine aminotransferase (ALT) 1063 U/L; aspartate aminotransferase (AST) 1772 U/L; C-reactive protein (CRP) 39 mg/L; white cell count (WCC) 14.4 × 10⁹/L; electrolytes and calcium were within normal limits. Urinalysis and ultrasound excluded obstructive uropathy. MRI of the lower limbs showed diffuse bilateral peri-muscular and soft tissue edema, suggesting myositis versus rhabdomyolysis (Figures 1-2). Subsequent EMG confirmed a myositis pattern without features of demyelinating polyneuropathy (Figure 3). 

Panels of autoimmune screens revealed ANA positivity, anti-Mi-2α and anti-PL-7 positivity, anti-HMGCR negativity, unremarkable viral and liver autoimmune serologies, and normal complement levels.

Nephrotoxic drugs and atorvastatin were instantly discontinued, and intravenous fluid was administered with close monitoring of her cardiac function. However, neither her renal function nor CK levels improved, and she required hemodialysis. Due to the presence of anti-Mi-2 with anti-PL-7 antibodies, she also commenced on high-dose intravenous methylprednisolone for immunosuppression, followed by a tapering course of oral prednisolone. The patient responded well to immunosuppressive therapy. Proximal muscle weakness improved significantly, and renal function started to recover. She was discharged after three weeks of admission with significant improvement in biochemical parameters, CK (189 U/L), and creatinine (232 µmol/L). She was followed up in the nephrology and rheumatology clinics post-discharge, and her renal function improved over the subsequent weeks (Table 1).

**Table 1:** Serial laboratory results, including CK and renal functions. CK, creatine kinase; WCC, white cell count; eGFR, estimated glomerular filtration rate; ESR, erythrocyte sedimentation rate; CRP, C-reactive protein; ALT, alanine aminotransferase; MCV, mean corpuscular volume; Cr, creatinine; Hb, hemoglobin

	ALP	ALT	Ca+	Albumin	CRP	CK	Cr	Hb	MCV	Neutrophils	PO4	Platelet	K+	Na+	Bilirubin	Urea	WCC	eGFR	ESR
19 Mar	243	1063	2.14	24	39	42322	262	165	98.8	7.04	1.57	182	4.2	133	52	18.8	10.71	15	-
20 Mar							329	166	100.8	8.65		204	4.3	135		20.4	14.14	11	-
21 Mar	198	806	2.17	17		29010	400	156	102.6	8.3	1.54	175	4.5	130	43	25.6	12.21	9	-
23 Mar	180	652		14	66	12085	468	148	100	8.95		164	5.1	131	42	31.4	11.52	7	-
24 Mar					72	13119	494	149	98.9	9.05		199	5	133		35.3	12.45	7	-
25 Mar	225	718	2.3	14	72	21488	543	153	97	9.34	2.49	202	5.4	135	36	37.9	12.61	6	-
26 Mar	224	718	2.27	16	77	15756	543		99.4	13.08	2.97	234	5.6	134	35	44	13.77	6	-
27 Mar	208		2.29	16	49		551		100	13.5	2.99	212	6.1	132		50.7	14.92	6	-
28 Mar	183		2.21	15	29	3862	546		97.4	10.44	3.13	194	4.9	132		53.5	11.39	6	-
29 Mar	191	487		16	19	2469	429	134	96.7	11.19		197	4.1	133	26	43.5	12.5	8	-
30 Mar	169	437		16		2046	320	131	97.7	11.6		128	4.1	133	29	30.7	14.22	12	-
31 Mar	163	367		16		1593	346	123	99.2	10.3		116	3.8	139	31	38.7	13.04	10	-
01 Apr	167	351	1.97	17	32	1694	372	125	100.5	11.09	2.05	122	4.5	141	35	42	14.11	10	-
02 Apr	136		1.98	16	29		374	105	100.3	9.79	2.12	110	3.3	142		43.1	12.44	9	-
03 Apr	152		2.18	19	25		352	111	100	9.92	2.04	118	3	143		44.8	12.67	10	-
04 Apr					20		333	115	101.8	8.56		134	3.2	145		43.7	11.55	11	-
07 Apr	145	184	2.53	24	14	189	284	108	106.6	10.83	1.26	144	4.9	151	27	39.6	13.26	13	-
09 Apr					15		232	105	105	8.72		133	3.8	138		31.9	9.44	17	-
16 May																			28
08 Jul																			18
10 Sep																			32

## Discussion

AIMs represent a clinically and immunologically heterogeneous spectrum of acquired muscle diseases characterized by immune-mediated skeletal muscle inflammation, elevated muscle enzymes, and frequently, extra-muscular organ involvement. This case underscores the critical diagnostic and therapeutic implications of recognizing AIM in patients presenting with profound rhabdomyolysis, acute kidney injury (AKI), and serological evidence of immune activation. The clinical scenario was initially complicated by several confounding factors, including chronic statin use and recent nitrofurantoin exposure, both of which have the potential to contribute to myopathy. However, the presence of disease-specific autoantibodies, marked steroid responsiveness, and imaging/electrophysiological findings ultimately favored autoimmune myositis over a purely toxic or statin-induced myopathy. Toxic statin myopathy and statin-triggered IMNM represent well-recognized clinical entities in elderly adults, especially in those with long-term statin exposure. Statins can cause a spectrum of myopathic presentations ranging from asymptomatic CK elevation to fulminant rhabdomyolysis. Statin-induced IMNM, typically associated with anti-HMGCR antibodies, is characterized by proximal muscle weakness, markedly elevated CK levels, and muscle fiber necrosis with minimal inflammation. The absence of anti-HMGCR antibodies in this patient, combined with the presence of inflammatory features on MRI and EMG, and seropositivity for myositis-specific autoantibodies, strongly argued against classic IMNM and favored AIM as the primary driver of pathology [3-5].

Moreover, the clinical phenotype of this patient extended beyond isolated muscle injury, with transaminitis and evidence of systemic inflammation, supporting an autoimmune rather than toxic etiology. While statins may act as potential precipitants or unmasking agents in susceptible individuals, their presence alone is insufficient to establish causality. Previous case reports have demonstrated that drug exposure often coexists with underlying immune-mediated conditions, which ultimately dictate disease trajectory and therapeutic response [7,8].

The detection of both anti-Mi-2α and anti-PL-7 antibodies in our patient provided clarification of the diagnosis and prognostic values. Anti-Mi-2 antibodies are classically associated with primary DM, typically presenting with proximal myopathy, characteristic cutaneous manifestations, and a favorable response to corticosteroids. Anti-Mi-2 positivity has also been linked to lower rates of interstitial lung disease (ILD) and overall better prognosis compared with other antibody subtypes [2-4]. In contrast, anti-PL-7, one of the antisynthetase antibodies, is typically associated with antisynthetase syndrome, which is a multisystem disorder characterized by myositis, ILD, arthritis, Raynaud phenomenon, mechanic’s hands, and systemic inflammation.

Double seropositivity for anti-Mi-2α and anti-PL-7 is rare but clinically significant. One study published a case describing similar dual positivity, highlighting the potential for overlapping clinical phenotypes with both good steroid response and increased risk of extra-muscular complications, including ILD [2]. Such dual autoantibody profiles may indicate a more complex and unpredictable clinical course, necessitating vigilant longitudinal monitoring and a low threshold for immunosuppressive therapy if systemic involvement develops.

In our patient, the absence of a cutaneous rash at presentation did not exclude DM, as up to 10%-20% of patients may manifest with a myositis-first or clinically amyopathic phenotype early in the disease evolution [3,4]. The combination of myositis-specific antibodies, MRI/EMG findings consistent with inflammatory myopathy, and rapid biochemical improvement with corticosteroid treatment all support AIM as the primary diagnosis.

Another key differential consideration was pure rhabdomyolysis secondary to drug-induced or metabolic injury. Both nitrofurantoin and statins have been associated with myocyte injury and transaminitis, and nitrofurantoin has been specifically linked to acute hepatitis, AKI, and elevated CK in rare cases [1]. However, the magnitude of CK elevation, antibody profile, inflammatory imaging positive findings, and favorable response to immunosuppression indicated that the rhabdomyolysis observed here was secondary to immune-mediated myositis rather than a primary drug-induced toxicity [1,5,6].

Compared to toxic rhabdomyolysis, autoimmune myositis often shows a prompt and sometimes dramatic clinical and biochemical response to corticosteroids, as was observed in this case. This rapid improvement is consistent with the anti-Mi-2-positive subset of DM, which is known to respond well to first-line immunosuppression [4].

A crucial diagnostic learning point from this case is the early differentiation between drug-associated and drug-induced disease. Nitrofurantoin is known to cause hepatic and renal injury, and both nitrofurantoin and statins are potential contributors to CK elevation. Previous case reports have highlighted the role of drug-drug interactions in amplifying statin toxicity, as seen with the co-administration of proton pump inhibitors and loop diuretics in a patient who developed DM and rhabdomyolysis. In that case, intravenous immunoglobulin (IVIG) was used as rescue therapy due to volume restrictions from renal failure [7]. Another report described DM induced by atorvastatin, which was refractory to corticosteroids and required rituximab for disease control, blurring the distinction between DM and IMNM [8].

In our patient, the negative anti-HMGCR antibody, double-positive anti-Mi-2α/PL-7 serology, and rapid corticosteroid responsiveness all strongly supported a primary autoimmune myositis rather than a statin-induced necrotizing myopathy. The medications likely acted as co-precipitants, lowering the threshold for immune activation or contributing to the severity of the myolysis.

This case represents three clinically actionable principles. First, AIM should remain high on the differential diagnosis in older adults presenting with massive CK elevation, rhabdomyolysis, and AKI, even when plausible drug triggers are present. Early testing for myositis-specific antibodies can significantly influence management decisions and lead to the initiation of immunosuppressive therapy, improving outcomes [3,4].

Second, rare dual seropositivity, such as anti-Mi-2α and anti-PL-7, may contribute to a more complex clinical trajectory. While anti-Mi-2 positivity often confers a good prognosis with rapid steroid responsiveness, coexisting antisynthetase antibodies can increase the risk of systemic complications, particularly ILD. Close respiratory monitoring and assessment, early pulmonary imaging tests, and long-term follow-up are therefore mandatory in this subgroup [2-4].

Third, the coincident drug exposure must be interpreted with caution. Drug-induced rhabdomyolysis remains an important diagnostic consideration; however, in many cases, including this one, drugs may act as non-specific triggers rather than primary pathological precipitants. The presence of specific autoantibodies and steroid responsiveness should prompt clinicians to prioritize immunosuppression, with escalation to agents such as IVIG or rituximab in refractory cases [7,8].

In contrast to previously reported cases where IVIG was required for steroid-refractory DM with rhabdomyolysis and AKI, our patient demonstrated substantial clinical improvement with corticosteroid monotherapy. This aligns with the favorable steroid-responsive phenotype typically associated with anti-Mi-2-positive DM [2,4].

In summary, this case highlights the importance of maintaining a high suspicious index for AIMs in patients with rhabdomyolysis and renal failure, even in the presence of potential drug triggers. The serological panels, particularly the rare double positivity for anti-Mi-2α and anti-PL-7, were pivotal in establishing the diagnosis and guiding treatment. Clinicians should recognize that medications may act as precipitating factors rather than solely cause such presentations. Early antibody testing, appropriate immunosuppression, and proactive systemic surveillance are key to optimizing patient outcomes.

## Conclusions

In older adults presenting with fulminant rhabdomyolysis and AKI, autoimmune myositis should remain front of mind even when statin exposure or adjunct antibiotics seem to *explain* the picture. Converging evidence from MRI/EMG and myositis-specific antibodies, including rare double positivity, can avoid misattribution to drug toxicity alone and prompt timely immunosuppression, improving outcomes. 
